# Supplementary material for: Exploring Connections between Oral Microbiota, Short-Chain Fatty Acids, and Specific Cancer Types: A Study of Oral Cancer, Head and Neck Cancer, Pancreatic Cancer, and Gastric Cancer
Source: Cancers (Basel). 2023 May 24;15(11):2898. doi: 10.3390/cancers15112898 (PMC10251859; doi:10.3390/cancers15112898)

Supplementary Content

Supplementary Table. S1 iSeq v4 fusion PCR primer

Forward Design	Sequence (89 mer)	P5 (29 mer)	i5 (8 mer)	NexTera concensus (14 mer)	Sequencing adaptor (19 mer)	Target region (515F)
S501	AATGATACGGCGACCACCGAGATCTACACTAGATCGCTCGTCGGCAGCGTCAGATGTGTATAAGAGACAGGTGCCAGCMGCCGCGGTAA	AATGATACGGCGACCACCGAGATCTACAC	TAGATCGC	TCGTCGGCAGCGTC	AGATGTGTATAAGAGACAG	GTGCCAGCMGCCGCGGTAA
S502	AATGATACGGCGACCACCGAGATCTACACCTCTCTATTCGTCGGCAGCGTCAGATGTGTATAAGAGACAGGTGCCAGCMGCCGCGGTAA	AATGATACGGCGACCACCGAGATCTACAC	CTCTCTAT	TCGTCGGCAGCGTC	AGATGTGTATAAGAGACAG	GTGCCAGCMGCCGCGGTAA
S503	AATGATACGGCGACCACCGAGATCTACACTATCCTCTTCGTCGGCAGCGTCAGATGTGTATAAGAGACAGGTGCCAGCMGCCGCGGTAA	AATGATACGGCGACCACCGAGATCTACAC	TATCCTCT	TCGTCGGCAGCGTC	AGATGTGTATAAGAGACAG	GTGCCAGCMGCCGCGGTAA
S505	AATGATACGGCGACCACCGAGATCTACACGTAAGGAGTCGTCGGCAGCGTCAGATGTGTATAAGAGACAGGTGCCAGCMGCCGCGGTAA	AATGATACGGCGACCACCGAGATCTACAC	GTAAGGAG	TCGTCGGCAGCGTC	AGATGTGTATAAGAGACAG	GTGCCAGCMGCCGCGGTAA
S506	AATGATACGGCGACCACCGAGATCTACACACTGCATATCGTCGGCAGCGTCAGATGTGTATAAGAGACAGGTGCCAGCMGCCGCGGTAA	AATGATACGGCGACCACCGAGATCTACAC	ACTGCATA	TCGTCGGCAGCGTC	AGATGTGTATAAGAGACAG	GTGCCAGCMGCCGCGGTAA
S507	AATGATACGGCGACCACCGAGATCTACACAAGGAGTATCGTCGGCAGCGTCAGATGTGTATAAGAGACAGGTGCCAGCMGCCGCGGTAA	AATGATACGGCGACCACCGAGATCTACAC	AAGGAGTA	TCGTCGGCAGCGTC	AGATGTGTATAAGAGACAG	GTGCCAGCMGCCGCGGTAA
S508	AATGATACGGCGACCACCGAGATCTACACCTAAGCCTTCGTCGGCAGCGTCAGATGTGTATAAGAGACAGGTGCCAGCMGCCGCGGTAA	AATGATACGGCGACCACCGAGATCTACAC	CTAAGCCT	TCGTCGGCAGCGTC	AGATGTGTATAAGAGACAG	GTGCCAGCMGCCGCGGTAA
S510	AATGATACGGCGACCACCGAGATCTACACCGTCTAATTCGTCGGCAGCGTCAGATGTGTATAAGAGACAGGTGCCAGCMGCCGCGGTAA	AATGATACGGCGACCACCGAGATCTACAC	CGTCTAAT	TCGTCGGCAGCGTC	AGATGTGTATAAGAGACAG	GTGCCAGCMGCCGCGGTAA
S511	AATGATACGGCGACCACCGAGATCTACACTCTCTCCGTCGTCGGCAGCGTCAGATGTGTATAAGAGACAGGTGCCAGCMGCCGCGGTAA	AATGATACGGCGACCACCGAGATCTACAC	TCTCTCCG	TCGTCGGCAGCGTC	AGATGTGTATAAGAGACAG	GTGCCAGCMGCCGCGGTAA
S513	AATGATACGGCGACCACCGAGATCTACACTCGACTAGTCGTCGGCAGCGTCAGATGTGTATAAGAGACAGGTGCCAGCMGCCGCGGTAA	AATGATACGGCGACCACCGAGATCTACAC	TCGACTAG	TCGTCGGCAGCGTC	AGATGTGTATAAGAGACAG	GTGCCAGCMGCCGCGGTAA
S515	AATGATACGGCGACCACCGAGATCTACACTTCTAGCTTCGTCGGCAGCGTCAGATGTGTATAAGAGACAGGTGCCAGCMGCCGCGGTAA	AATGATACGGCGACCACCGAGATCTACAC	TTCTAGCT	TCGTCGGCAGCGTC	AGATGTGTATAAGAGACAG	GTGCCAGCMGCCGCGGTAA
S516	AATGATACGGCGACCACCGAGATCTACACCCTAGAGTTCGTCGGCAGCGTCAGATGTGTATAAGAGACAGGTGCCAGCMGCCGCGGTAA	AATGATACGGCGACCACCGAGATCTACAC	CCTAGAGT	TCGTCGGCAGCGTC	AGATGTGTATAAGAGACAG	GTGCCAGCMGCCGCGGTAA
S517	AATGATACGGCGACCACCGAGATCTACACGCGTAAGATCGTCGGCAGCGTCAGATGTGTATAAGAGACAGGTGCCAGCMGCCGCGGTAA	AATGATACGGCGACCACCGAGATCTACAC	GCGTAAGA	TCGTCGGCAGCGTC	AGATGTGTATAAGAGACAG	GTGCCAGCMGCCGCGGTAA
S518	AATGATACGGCGACCACCGAGATCTACACCTATTAAGTCGTCGGCAGCGTCAGATGTGTATAAGAGACAGGTGCCAGCMGCCGCGGTAA	AATGATACGGCGACCACCGAGATCTACAC	CTATTAAG	TCGTCGGCAGCGTC	AGATGTGTATAAGAGACAG	GTGCCAGCMGCCGCGGTAA
S520	AATGATACGGCGACCACCGAGATCTACACAAGGCTATTCGTCGGCAGCGTCAGATGTGTATAAGAGACAGGTGCCAGCMGCCGCGGTAA	AATGATACGGCGACCACCGAGATCTACAC	AAGGCTAT	TCGTCGGCAGCGTC	AGATGTGTATAAGAGACAG	GTGCCAGCMGCCGCGGTAA
S521	AATGATACGGCGACCACCGAGATCTACACGAGCCTTATCGTCGGCAGCGTCAGATGTGTATAAGAGACAGGTGCCAGCMGCCGCGGTAA	AATGATACGGCGACCACCGAGATCTACAC	GAGCCTTA	TCGTCGGCAGCGTC	AGATGTGTATAAGAGACAG	GTGCCAGCMGCCGCGGTAA
S522	AATGATACGGCGACCACCGAGATCTACACTTATGCGATCGTCGGCAGCGTCAGATGTGTATAAGAGACAGGTGCCAGCMGCCGCGGTAA	AATGATACGGCGACCACCGAGATCTACAC	TTATGCGA	TCGTCGGCAGCGTC	AGATGTGTATAAGAGACAG	GTGCCAGCMGCCGCGGTAA

Reverse Design	Sequence (86 mer)	P7 (24 mer)	i7 (8 mer)	NexTera concensus (15 mer)	Sequencing adaptor (19 mer)	Target region (806R)
N701	CAAGCAGAAGACGGCATACGAGATTCGCCTTAGTCTCGTGGGCTCGGAGATGTGTATAAGAGACAGGGACTACHVGGGTWTCTAAT	CAAGCAGAAGACGGCATACGAGAT	TCGCCTTA	GTCTCGTGGGCTCGG	AGATGTGTATAAGAGACAG	GGACTACHVGGGTWTCTAAT
N702	CAAGCAGAAGACGGCATACGAGATCTAGTACGGTCTCGTGGGCTCGGAGATGTGTATAAGAGACAGGGACTACHVGGGTWTCTAAT	CAAGCAGAAGACGGCATACGAGAT	CTAGTACG	GTCTCGTGGGCTCGG	AGATGTGTATAAGAGACAG	GGACTACHVGGGTWTCTAAT
N703	CAAGCAGAAGACGGCATACGAGATTTCTGCCTGTCTCGTGGGCTCGGAGATGTGTATAAGAGACAGGGACTACHVGGGTWTCTAAT	CAAGCAGAAGACGGCATACGAGAT	TTCTGCCT	GTCTCGTGGGCTCGG	AGATGTGTATAAGAGACAG	GGACTACHVGGGTWTCTAAT
N704	CAAGCAGAAGACGGCATACGAGATGCTCAGGAGTCTCGTGGGCTCGGAGATGTGTATAAGAGACAGGGACTACHVGGGTWTCTAAT	CAAGCAGAAGACGGCATACGAGAT	GCTCAGGA	GTCTCGTGGGCTCGG	AGATGTGTATAAGAGACAG	GGACTACHVGGGTWTCTAAT
N705	CAAGCAGAAGACGGCATACGAGATAGGAGTCCGTCTCGTGGGCTCGGAGATGTGTATAAGAGACAGGGACTACHVGGGTWTCTAAT	CAAGCAGAAGACGGCATACGAGAT	AGGAGTCC	GTCTCGTGGGCTCGG	AGATGTGTATAAGAGACAG	GGACTACHVGGGTWTCTAAT
N706	CAAGCAGAAGACGGCATACGAGATCATGCCTAGTCTCGTGGGCTCGGAGATGTGTATAAGAGACAGGGACTACHVGGGTWTCTAAT	CAAGCAGAAGACGGCATACGAGAT	CATGCCTA	GTCTCGTGGGCTCGG	AGATGTGTATAAGAGACAG	GGACTACHVGGGTWTCTAAT
N707	CAAGCAGAAGACGGCATACGAGATGTAGAGAGGTCTCGTGGGCTCGGAGATGTGTATAAGAGACAGGGACTACHVGGGTWTCTAAT	CAAGCAGAAGACGGCATACGAGAT	GTAGAGAG	GTCTCGTGGGCTCGG	AGATGTGTATAAGAGACAG	GGACTACHVGGGTWTCTAAT
N710	CAAGCAGAAGACGGCATACGAGATCAGCCTCGGTCTCGTGGGCTCGGAGATGTGTATAAGAGACAGGGACTACHVGGGTWTCTAAT	CAAGCAGAAGACGGCATACGAGAT	CAGCCTCG	GTCTCGTGGGCTCGG	AGATGTGTATAAGAGACAG	GGACTACHVGGGTWTCTAAT
N711	CAAGCAGAAGACGGCATACGAGATTGCCTCTTGTCTCGTGGGCTCGGAGATGTGTATAAGAGACAGGGACTACHVGGGTWTCTAAT	CAAGCAGAAGACGGCATACGAGAT	TGCCTCTT	GTCTCGTGGGCTCGG	AGATGTGTATAAGAGACAG	GGACTACHVGGGTWTCTAAT
N712	CAAGCAGAAGACGGCATACGAGATTCCTCTACGTCTCGTGGGCTCGGAGATGTGTATAAGAGACAGGGACTACHVGGGTWTCTAAT	CAAGCAGAAGACGGCATACGAGAT	TCCTCTAC	GTCTCGTGGGCTCGG	AGATGTGTATAAGAGACAG	GGACTACHVGGGTWTCTAAT
N714	CAAGCAGAAGACGGCATACGAGATTCATGAGCGTCTCGTGGGCTCGGAGATGTGTATAAGAGACAGGGACTACHVGGGTWTCTAAT	CAAGCAGAAGACGGCATACGAGAT	TCATGAGC	GTCTCGTGGGCTCGG	AGATGTGTATAAGAGACAG	GGACTACHVGGGTWTCTAAT
N715	CAAGCAGAAGACGGCATACGAGATCCTGAGATGTCTCGTGGGCTCGGAGATGTGTATAAGAGACAGGGACTACHVGGGTWTCTAAT	CAAGCAGAAGACGGCATACGAGAT	CCTGAGAT	GTCTCGTGGGCTCGG	AGATGTGTATAAGAGACAG	GGACTACHVGGGTWTCTAAT
N716	CAAGCAGAAGACGGCATACGAGATTAGCGAGTGTCTCGTGGGCTCGGAGATGTGTATAAGAGACAGGGACTACHVGGGTWTCTAAT	CAAGCAGAAGACGGCATACGAGAT	TAGCGAGT	GTCTCGTGGGCTCGG	AGATGTGTATAAGAGACAG	GGACTACHVGGGTWTCTAAT
N718	CAAGCAGAAGACGGCATACGAGATGTAGCTCCGTCTCGTGGGCTCGGAGATGTGTATAAGAGACAGGGACTACHVGGGTWTCTAAT	CAAGCAGAAGACGGCATACGAGAT	GTAGCTCC	GTCTCGTGGGCTCGG	AGATGTGTATAAGAGACAG	GGACTACHVGGGTWTCTAAT
N719	CAAGCAGAAGACGGCATACGAGATTACTACGCGTCTCGTGGGCTCGGAGATGTGTATAAGAGACAGGGACTACHVGGGTWTCTAAT	CAAGCAGAAGACGGCATACGAGAT	TACTACGC	GTCTCGTGGGCTCGG	AGATGTGTATAAGAGACAG	GGACTACHVGGGTWTCTAAT
N720	CAAGCAGAAGACGGCATACGAGATAGGCTCCGGTCTCGTGGGCTCGGAGATGTGTATAAGAGACAGGGACTACHVGGGTWTCTAAT	CAAGCAGAAGACGGCATACGAGAT	AGGCTCCG	GTCTCGTGGGCTCGG	AGATGTGTATAAGAGACAG	GGACTACHVGGGTWTCTAAT
N721	CAAGCAGAAGACGGCATACGAGATGCAGCGTAGTCTCGTGGGCTCGGAGATGTGTATAAGAGACAGGGACTACHVGGGTWTCTAAT	CAAGCAGAAGACGGCATACGAGAT	GCAGCGTA	GTCTCGTGGGCTCGG	AGATGTGTATAAGAGACAG	GGACTACHVGGGTWTCTAAT
N722	CAAGCAGAAGACGGCATACGAGATCTGCGCATGTCTCGTGGGCTCGGAGATGTGTATAAGAGACAGGGACTACHVGGGTWTCTAAT	CAAGCAGAAGACGGCATACGAGAT	CTGCGCAT	GTCTCGTGGGCTCGG	AGATGTGTATAAGAGACAG	GGACTACHVGGGTWTCTAAT
N723	CAAGCAGAAGACGGCATACGAGATGAGCGCTAGTCTCGTGGGCTCGGAGATGTGTATAAGAGACAGGGACTACHVGGGTWTCTAAT	CAAGCAGAAGACGGCATACGAGAT	GAGCGCTA	GTCTCGTGGGCTCGG	AGATGTGTATAAGAGACAG	GGACTACHVGGGTWTCTAAT
N724	CAAGCAGAAGACGGCATACGAGATCGCTCAGTGTCTCGTGGGCTCGGAGATGTGTATAAGAGACAGGGACTACHVGGGTWTCTAAT	CAAGCAGAAGACGGCATACGAGAT	CGCTCAGT	GTCTCGTGGGCTCGG	AGATGTGTATAAGAGACAG	GGACTACHVGGGTWTCTAAT
N726	CAAGCAGAAGACGGCATACGAGATGTCTTAGGGTCTCGTGGGCTCGGAGATGTGTATAAGAGACAGGGACTACHVGGGTWTCTAAT	CAAGCAGAAGACGGCATACGAGAT	GTCTTAGG	GTCTCGTGGGCTCGG	AGATGTGTATAAGAGACAG	GGACTACHVGGGTWTCTAAT
N727	CAAGCAGAAGACGGCATACGAGATACTGATCGGTCTCGTGGGCTCGGAGATGTGTATAAGAGACAGGGACTACHVGGGTWTCTAAT	CAAGCAGAAGACGGCATACGAGAT	ACTGATCG	GTCTCGTGGGCTCGG	AGATGTGTATAAGAGACAG	GGACTACHVGGGTWTCTAAT
N728	CAAGCAGAAGACGGCATACGAGATTAGCTGCAGTCTCGTGGGCTCGGAGATGTGTATAAGAGACAGGGACTACHVGGGTWTCTAAT	CAAGCAGAAGACGGCATACGAGAT	TAGCTGCA	GTCTCGTGGGCTCGG	AGATGTGTATAAGAGACAG	GGACTACHVGGGTWTCTAAT
N729	CAAGCAGAAGACGGCATACGAGATGACGTCGAGTCTCGTGGGCTCGGAGATGTGTATAAGAGACAGGGACTACHVGGGTWTCTAAT	CAAGCAGAAGACGGCATACGAGAT	GACGTCGA	GTCTCGTGGGCTCGG	AGATGTGTATAAGAGACAG	GGACTACHVGGGTWTCTAAT

Supplementary Table. S2 General characteristics of 745 control and 178 OC, 21 HNC, 50 PC, 60 GC patients

Variable	Control (n=745)	OC (n=178)	HNC (n=21)	PC (n=50)	GC (n=60)	*P* value[a]
**Age**[a]	57.3 ± 9.2	65.1 ± 13.4	57.7 ± 13.0	63.0 ± 9.2	59.8 ± 9.1	<.0001
**Sex**[b]						<.0001
F	434 (58.3%)	73 (41.0%)	5 (23.8%)	23 (46.0%)	15 (25.0%)	
M	311 (41.7%)	105 (59.0%)	16 (76.2%)	27 (54.0%)	45 (75.0%)	
**BMI**[c]	24.0 ± 3.0	23.7 ± 3.9	24.6 ± 4.5	22.1 ± 2.7	24.3 ± 3.2	0.001
<18.5	17 (2.3%)	21 (11.8%)	0 (0.0%)	3 (6.0%)	0 (0.0%)	<.0001
18.5~22.9	272 (36.5%)	48 (27.0%)	6 (28.6%)	28 (56.0%)	19 (31.7%)	
23.0~24.9	197 (26.4%)	39 (21.9%)	6 (28.6%)	10 (20.0%)	23 (38.3%)	
25.0~29.9	226 (30.3%)	59 (33.2%)	7 (33.3%)	9 (18.0%)	15 (25.0%)	
30≥	27 (3.6%)	7 (3.9%)	1 (4.8%)	0 (0.0%)	3 (5.0%)	
**Smoke**[d]						<.0001
Non smoker	447 (60.0%)	95 (53.4%)	6 (28.6%)	26 (52.0%)	25 (41.7%)	
Former smoker	202 (27.1%)	45 (25.3%)	9 (42.9%)	14 (28.0%)	17 (28.3%)	
Current smoker	71 (9.5%)	33 (18.5%)	6 (28.6%)	10 (20.0%)	17 (28.3%)	
**Drink**[e]						<.0001
Non drinker	179 (24.0%)	82 (46.1%)	3 (14.3%)	19 (38.0%)	14 (23.3%)	
Former drinker	85 (11.4%)	28 (15.7%)	8 (38.1%)	11 (22.0%)	9 (15.0%)	
Current drinker	441 (59.2%)	63 (35.4%)	10 (47.6%)	20 (40.0%)	36 (60.0%)	
**Stage**[f]						<.0001
1		34 (19.1%)	6 (28.6%)	2 (4.0%)	47 (78.3%)	
2		29 (16.3%)	4 (19.1%)	6 (12.0%)	4 (6.7%)	
3		29 (16.3%)	2 (9.5%)	7 (14.0%)	5 (8.3%)	

4	72 (40.5%)	2 (9.5%)	25 (50.0%)	2 (3.3%)	
**T Stage**[g]					<.0001
T1	35 (19.7%)	9 (42.9%)	5 (10.0%)	47 (78.3%)	
T2	41 (23.0%)	5 (23.8%)	7 (14.0%)	4 (6.7%)	
T3	28 (15.7%)	3 (14.3%)	2 (4.0%)	3 (5.0%)	
T4	57 (32.0%)	2 (9.5%)	0 (0.0%)	4 (6.7%)	
**N stage**[h]					<.0001
N0	107 (60.1%)	5 (23.8%)	6 (12.0%)	23 (38.3%)	
N1	21 (11.8%)	8 (38.1%)	5 (10.0%)	6 (10.0%)	
N2	21 (11.8%)	4 (19.1%)	3 (6.0%)	4 (6.7%)	
N3	12 (6.7%)	1 (4.8%)	0 (0.0%)	1 (1.7%)	

a p value from t-test for continuous variables and from chi-square test for categorical variables for comparison between all groups. between all groups. c, BMI was categorized into 5 groups, there are underweight people with less than 18.5, normal range people with 18.5 to 22.9, overweight people with 23.0 to 24.9, obese people with 25.0 to 29.9, and highly obese people with a BMI of 30 or more. d, Smoking status was categorized into 3 groups, those who never smoked, former smokers, current smokers. e, drinking status was categorized into 3 groups, those who never drink, former drinker, current drinker. f, Stage information were collected from cancer patients except for the control group. g, T stage information in the GC patients was not collected, only 229 cancer patients were included. h, N stage information in the PC, GC patients was not collected, only 170 cancer patients were included. BMI, Body mass index. T stage, Tumor stage. N stage, Node stage.

Supplementary Table. S3 Association between oral cancer risk and 6 genera with age and sex.

	Age < 60		Age >60	
	Cancer (n=191) / Control (n=460)		Cancer (n=118) / Control (n=258)	
	Continuous logistic regression[a]	P value[b]	Continuous logistic regression[a]	P value[b]
Streptococcus	1.76 (0.98-3.17)	5.82	2.39 (1.75-3.26)	4.88
Haemophilus	2.31 (1.07-4.97)	3.32	2.34 (1.22-4.48)	1.02
Prevotella	0.53 (0.32-0.87)	1.2E-02	0.47 (0.35-0.62)	6.1.E-08
Leuconostoc	3.92 (2.01-7.64)	6.25	3.07 (1.94-4.85)	1.56
Neisseria	0.35 (0.25-0.48)	3.0E-10	0.49 (0.42-0.58)	1.3.E-17
Abiotrophia	2.11 (1.43-3.10)	1.54	1.35 (1.12-1.63)	1. 03

	Sex: Female		Sex: Male	
	Cancer (n=55) / Control (n=870)		Cancer (n=94) / Control (n=870)	
	Continuous logistic regression[a]	P value[b]	Continuous logistic regression[a]	P value[b]
Streptococcus	2.17 (1.18-4.02)	0.13	2.39 (1.25-4.56)	3.23
Haemophilus	1.78 (1.06-2.97)	2.8.E-02	3.82 (2.45-5.95)	3.2.E-09
Prevotella	0.60 (0.50-0.73)	4.27	0.50 (0.43-0.58)	1.33
Leuconostoc	1.56 (1.31-1.86)	0.02	1.64 (1.43-1.89)	0.05
Neisseria	0.39 (0.29-0.53)	1.3.E-09	0.45 (0.35-0.58)	5.1.E-10
Abiotrophia	3.36 (2.33-4.85)	1.09	2.45 (1.84-3.26)	2.76
Streptococcus	1.76 (1.40-2.23)	2.34	1.47 (1.24-1.75)	0.23

[a]Continuous logistic regression of 6 genera. [b]P value was computed using chi-sqaure test for continuous scale.

Supplementary Table. S4 Comparative Analysis of 6 genera among cancer and control

Taxon Name	Abundance Quartile [a]						Fold Change [b] Median(cancer) / Median(control)	Wilcoxon rank sum p-value [c]
	Control			Cancer				
	Q1	Q2 (Median)	Q3	Q1	Q2 (Median)	Q3		
Streptococcus	3.0407	4.7543	7.3595	5.2413	8.3496	14.9677	1.7562	5.24E-26
Haemophilus	6.7991	10.7951	15.4098	2.4379	6.0654	12.6851	0.5619	2.79E-15
Prevotella	10.1393	17.0575	27.0389	6.2372	11.5618	21.2336	0.6778	2.18E-10
Leuconostoc	0	0	0	0	0	0.0032	NA	2.82E-39
Neisseria	7.4227	14.8937	24.946	4.2272	11.4618	22.7215	0.7696	0.000265546
Abiotrophia	0	0.002	0.0142	0	0.006	0.0745	3	1.18E-09

a, Abundance Quartile: Q1(=25% percentile=0.25 quantile), Q2(=median =50% percentile =0.50 quantile), Q3(=75% percentile =0.75 quantile). b, Median value of the cancer divided by the median value of the controls. c, p value was computed using Wilcoxon rank-sum test for continuous variables. same with Mann Whitney U test.

Supplementary Fig. S1 Average composition of bacterial community at phylum levels with the relative abundance greater than 1%.

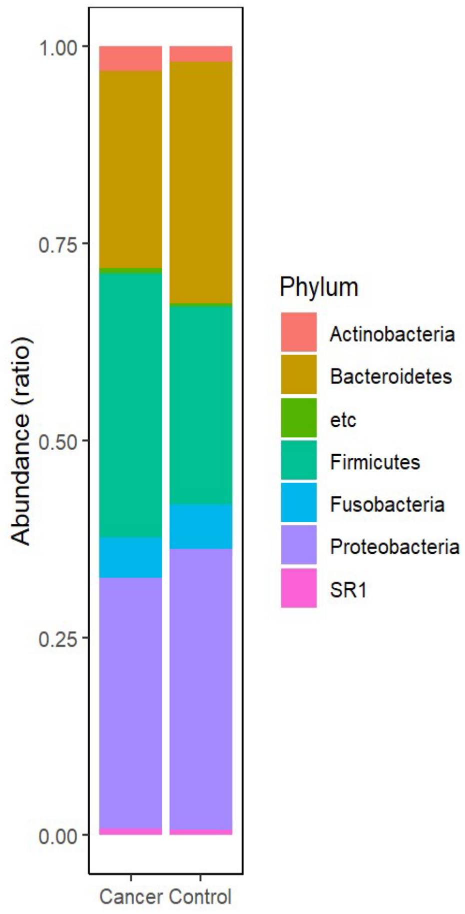

Supplementary Fig. S2 LEfSe analysis cladogram of the oral microbiota in control and cancer groups

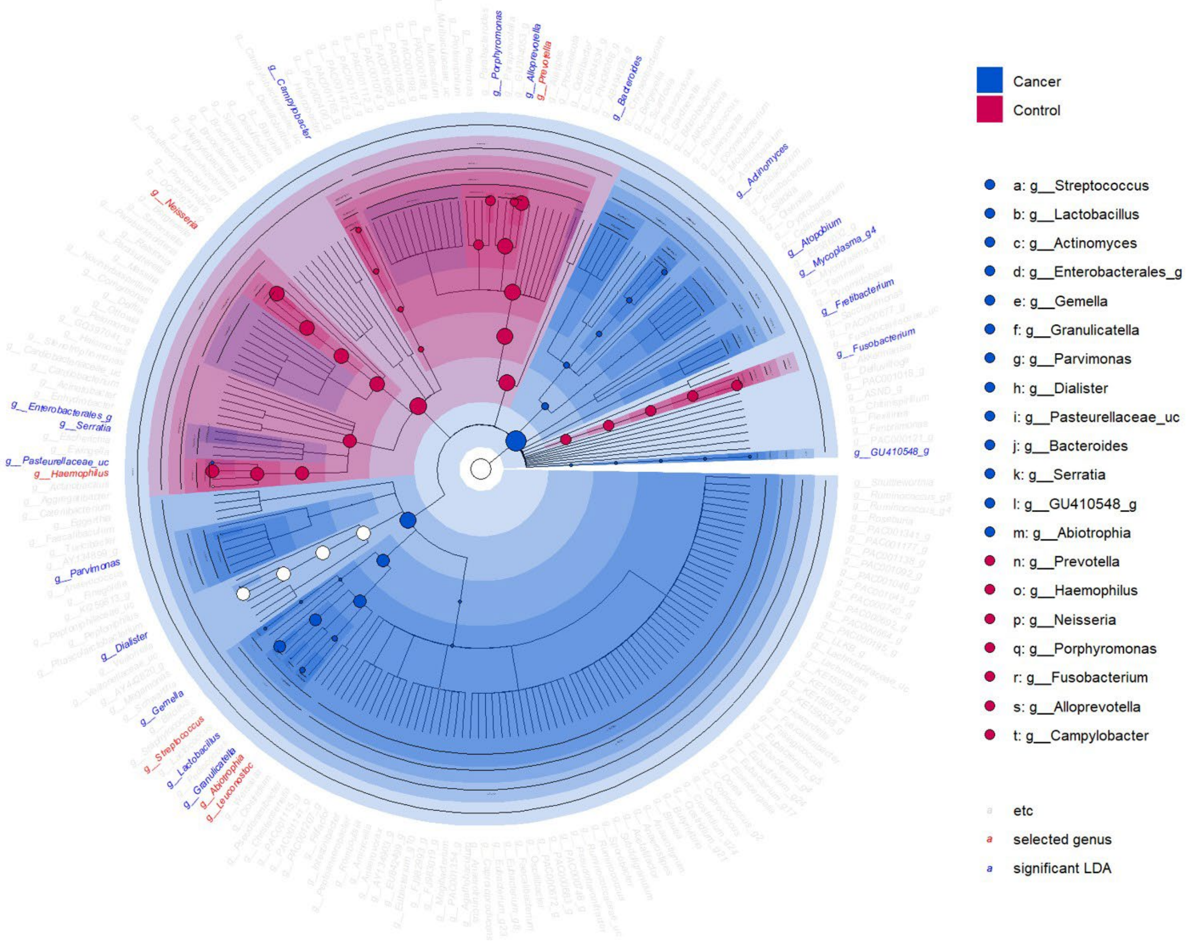

Supplementary Fig. S3 Association between 6 genera of oral microbiota with smoking status

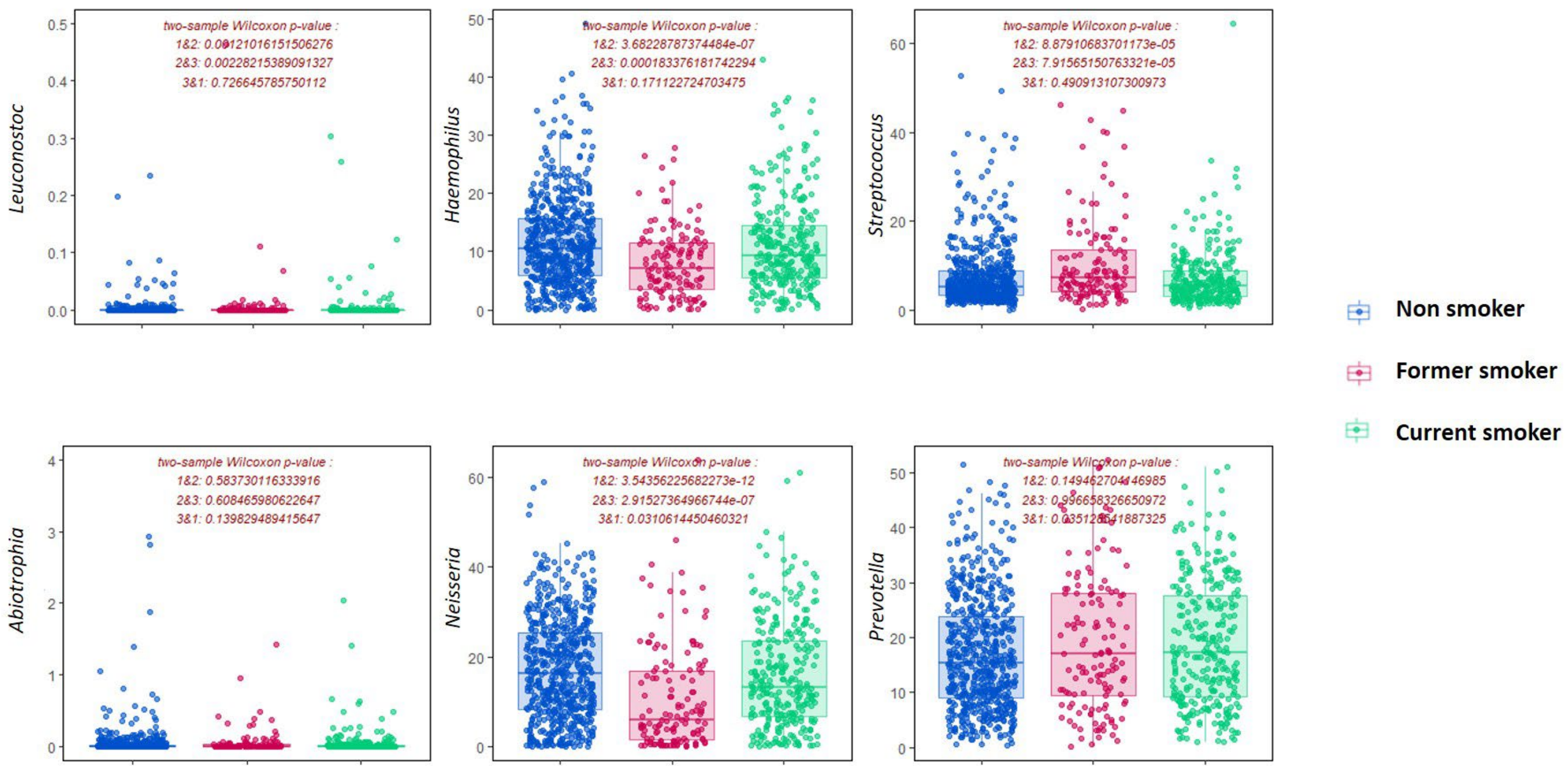

Supplementary Fig. S4 Association between 6 genera of oral microbiota with drinking status

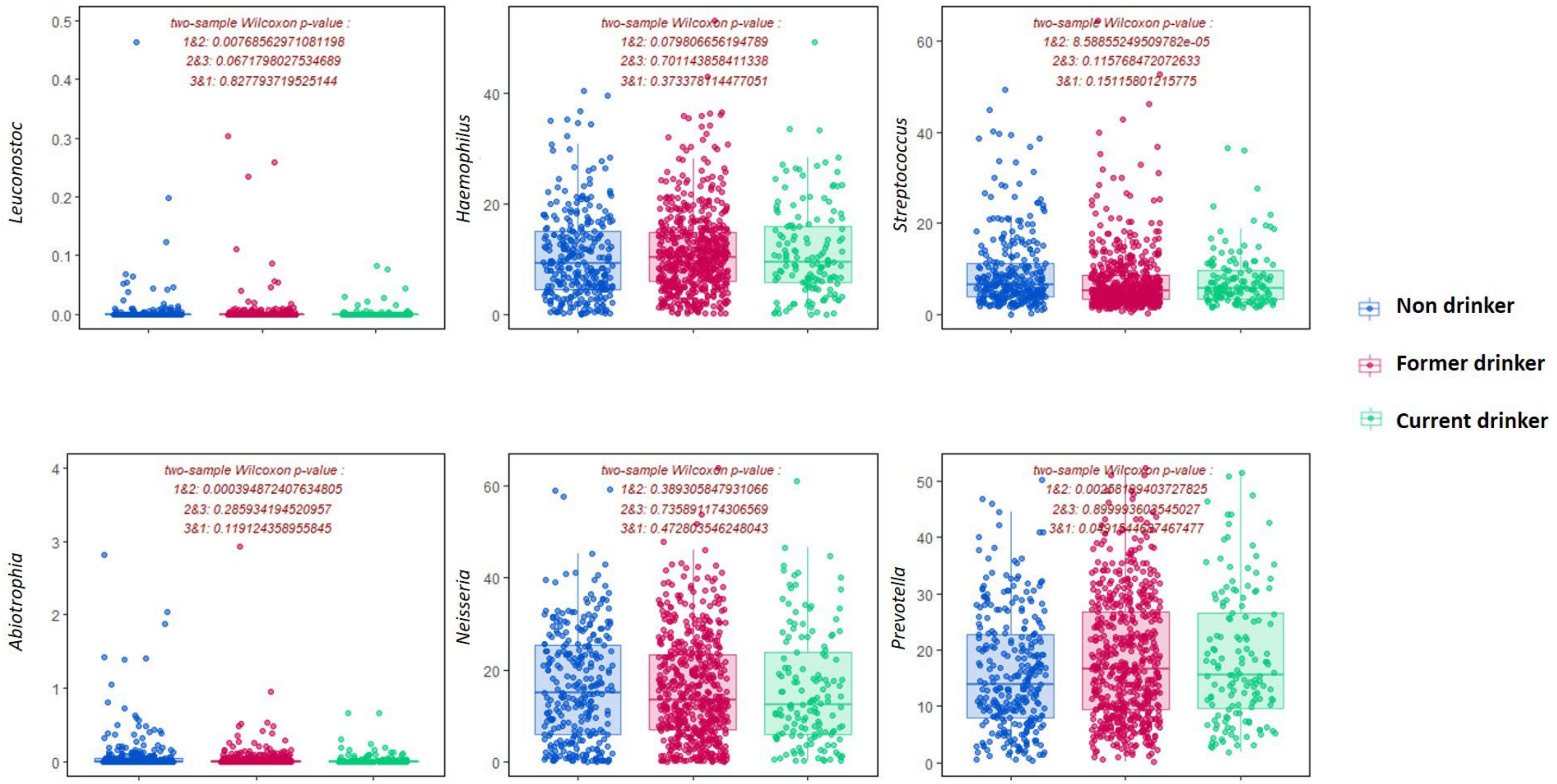

Supplement: Supplementary file 1 [file cancers-15-02898-s001.zip › cancers-2340006-supplementary.pdf]
